# Overall Survival Analyses following Adjuvant Chemotherapy or Nonadjuvant Chemotherapy in Patients with Stage IB Non-Small-Cell Lung Cancer

**DOI:** 10.1155/2021/8052752

**Published:** 2021-07-17

**Authors:** Zegui Tu, Tian Tian, Qian Chen, Caili Li

**Affiliations:** ^1^Department of Thoracic Oncology, West China Hospital of Sichuan University, Chengdu 610041, Sichuan, China; ^2^West China Medical School, Sichuan University, Chengdu 610041, Sichuan, China; ^3^Department of Clinical Research Management, West China Hospital of Sichuan University, Chengdu 610041, Sichuan, China; ^4^West China School of Nursing, Sichuan University, Chengdu 610041, Sichuan, China

## Abstract

**Background:**

Adjuvant chemotherapy (ACT) can improve prognosis for stages II-IIIA patients with non-small-cell lung cancer (NSCLC), but its implication in stage I patients is still an intractable puzzle. This study aims to seek ACT candidates for stage IB NSCLC and establish a nomogram to predict overall survival (OS) of specific patient for clinician's decision.

**Method:**

We performed a retrospective study on 16,765 patients (ACT group: *n* = 2,187; non-ACT group: *n* = 14,578) from the Surveillance, Epidemiology, and End Results (SEER) database. Overall survival was assessed in two groups. We performed propensity-score matching for risk adjustment. The risk factors were identified and used to create nomogram. Concordance index (C-index), Hosmer–Lemeshow test, and calibration were applied to evaluate model performance. To further evaluate the influence of tumor size on the selection of potential ACT candidates for patients with stage IB NSCLC, subgroup analyses were executed.

**Result:**

Survival analysis for the entire study cohort showed that ACT had better OS than non-ACT (HR = 0.800, CI: (0.751–0.851), *P* < 0.0001). In matched cohort, ACT also presented better OS than non-ACT (HR = 0.775, CI: (0.704–0.853), *P* < 0.0001). Univariate and multivariate Cox regression analysis revealed that eight prognostic factors, including gender, age, grade, pathological subtype, tumor size, visceral pleural invasion, surgical procedure, and the number of removed lymph nodes, were significantly correlated with OS. The nomogram was further constructed based on these prognostic factors. The C-index of nomogram was 0.639 (95%CI: 0.632–0.646). The Hosmer–Lemeshow test, and calibration presented good congruence between the predictions and actual observations. Subgroup analyses of tumor size group showed that ACT shared similar OS to non-ACT in NSCLC patients with tumor size ≤20 mm (*P* > 0.05). However, for NSCLC patients with 20 mm < size ≤30 mm (HR = 0.845, 95%CI (0.724–0.986), *P*=0.032) and 30 mm < size ≤40 mm (HR = 0.912, 95%CI (0.833–1.000), *P*=0.049), ACT associated with better OS.

**Conclusion:**

In this study, we found that ACT had better OS than non-ACT in patients with stage IB NSCLC. The nomogram provided an individual prediction of OS for patients after surgical resection. Patients with tumor size >20 mm and ≤40 mm may be potential candidates for ACT.

## 1. Background

Lung cancer has been more prevalent and the burden of disease became huge in developed countries' health systems [1]. Nearly 85% of all cases are non-small-cell lung cancer (NSCLC), and the most common histological subtypes are adenocarcinoma and squamous cell carcinoma [2, 3]. For stage IB NSCLC patients, complete surgical resection remains optimal choice [4]. However, 18%–29% patients recurred and died within 5 years after resection [5]. Even the 8th edition TNM staging system excludes tumors >4 cm from stage IB NSCLC population, and the 5-year overall survival (OS) is still 80% [6, 7].

Postoperative adjuvant chemotherapy (ACT), a member of comprehensive therapy in cancer treatment, can reduce the onset of recurrence and metastasis for patients [8]. Many large, randomized, phase III trials showed that ACT can improve prognosis for stages II-IIIA patients with NSCLC, but its implication in stage I patients is still an intractable puzzle [9–11]. A meta-analysis showed ACT can effectively improve OS for stage IB NSCLC patients, whereas some randomized controlled studies presented different results [12–14]. Moreover, due to discord between older TNM staging systems and 8th TNM staging system (tumor size >4 cm but ≤5 cm with node-negative has been classified as stage IIA instead of stage IB in 8th TNM staging system), many results from previous studies may not be accurate in stage IB NSCLC now [6]. Therefore, it is urgently needed to redefine ACT candidates for current stage IB patients.

Some studies have proposed that patients with high-risk factors would be better candidate for ACT, because they may have a high incidence of recurrence [15–17]. Poorly differentiated tumors, vascular invasion, visceral pleural involvement (VPI), unknown lymph node status, and wedge resection are recognized as high-risk factors in stage IB patients under the latest National Comprehensive Cancer Network (NCCN) guideline. Nevertheless, it is unknown whether there is a risk difference between them, and other factors not included in the guideline also present influence in prognosis [18, 19]. The NCCN guideline also states that high-risk factors are not an absolute indication for ACT in stage IB. So, this raises confusion in clinical practice, as the use of ACT relies on the clinician's judgement. Given there is insufficient information regarding the need for adjuvant chemotherapy in such patients, nomogram would be a better approach to solute this dilemma. Nomogram as a comprehensive assessing model can qualify relevant risk factors and provide a total point which can predict the survival of patients [20]. Clinician can easily make a judgement for specific patient by using it to evaluate patients' survival. Many studies also proved that nomogram had better predictive power than traditional TNM staging systems [21, 22]. Nevertheless, for stage IB NSCLC patients, nomograms to predict survival outcomes are scarce. In our study, we conducted overall survival analyses following adjuvant chemotherapy or nonadjuvant chemotherapy in patients with stage IB non-small-cell lung cancer to seek ACT candidates and construct a nomogram for clinician's decision.

## 2. Methods

### 2.1. Study Population and Selection Criteria

Clinical information for stage IB NSCLC patients between 2004 and 2015 was extracted from the Surveillance, Epidemiology, and End Results (SEER) database (http://seer.cancer.gov/). The inclusion criteria consisted of the following: (1) pathological diagnosis being NSCLC, (2) T2aN0M0 stage tumor according to 8th edition TNM staging system classification (tumor size >30 mm and ≤40 mm or tumor size ≤30 mm with visceral pleural involvement), and (3) surgical history of lobectomy, segmentectomy, or wedge resection. We excluded patients including the following: (1) history of radiotherapy; (2) age <20; (3) no information about history of chemotherapy and accurate follow-up on extracted data.

The patient's demographics (age, gender, race, and material status), features of tumors (size, status of visceral pleural invasion, laterality, differentiation grade, and pathological subtype), treatment details (year of diagnosis, surgical type, the number of removed lymph nodes, and history of chemotherapy), and follow-up details (survival status and survival time) were extracted from the SEER. We classified patients receiving ACT as ACT group and patients without ACT as non-ACT group. OS was primary endpoint in our analyses.

### 2.2. Statistical Analysis

To maximize the representativeness of our study, we extracted data using SEER 18 databases (with additional treatment fields, Nov 2018 sub). The characteristics of the study were summarized by using Pearson's Chi square test. Kaplan–Meier method was applied to assess survival curve of OS, and the significance was assessed by the log-rank test. Propensity-score matching was applied to mitigate potential bias at baseline between groups. The non-ACT matched in a 1 : 1 ratio to ACT based on the propensity score with a standard caliper width of 0.05. After matching, the degree of baseline variable balance was assessed by standardized differences. A standardized difference of 0.1 reflects high degree of balance. The independent prognostic factors of OS were evaluated by applying univariate and multivariate Cox regression analyses in the non-ACT group. Multivariable analyses were performed in a forward stepwise manner and hazards ratios (HR), and 95% confidence intervals (95% CI) were calculated.

According to the results of the multivariable analyses in the cohort of non-ACT, a nomogram was generated and provided an opportunity to calculate the probability of OS at 48, 96, and 144 months. Concordance index (C-index), Hosmer–Lemeshow test, and calibration was used to assess model performance. Calibration of the nomogram for 48-, 96-, and 144-month OS was conducted by comparing the prediction with observation in the probability of OS.

To further evaluate the influence of tumor size on the selection of potential ACT candidates for patients with stage IB NSCLC, Hazard ratio (HR) and 95% confidence intervals (95% CI) were computed by using multivariate Cox regression analyses.

SPSS Software (version 26.0; IBM Corporation) was applied for statistical analysis. Kaplan–Meier survival curve was formulated using GraphPad Prism 8 (version 8.0.1; GraphPad Software). R software (version 4.0.2; http://www.r-project.org) with packages of survival, rms, MatchIt, and ResourceSelection was used to establish nomogram and statistical analysis. *P* < 0.05 was statistically significant, and all statistical tests were two-tailed.

## 3. Results

### 3.1. Clinicopathological Characteristics of Study Cohort

From 2004 to 2015, 16,765 patients with NSCLC from SEER database who were pathologically diagnosed to be stage T2aN0M0 were included. Among these patients, 14,578 underwent non-ACT and 2,187 ACT. The age of the entire cohort spanned from 20 to 85+, and the median age was 72. The median of OS was 53.31 months and interquartile range (IQR) of OS from 25.00 months to 76.00 months.  Table 1 enumerated all baseline characteristics of overall cohort. The results from Table 1 showed that patients receiving ACT tended to be younger, married, with right lung lesions, with advanced tumor grade, with adenocarcinoma, with larger tumor size, and with lobectomy (*P* < 0.05).

### 3.2. Survival Analysis of Entire Study Cohort and Matched Study Cohort

**Figure 1:**
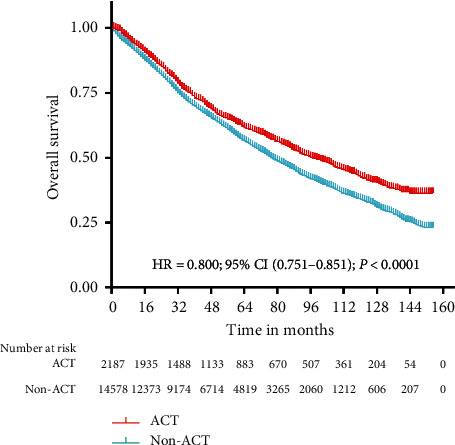
Overall survival for the entire study cohort. The Kaplan–Meier survival analysis for entire study cohort showed that ACT had better OS than non-ACT (HR = 0.800, CI: (0.751–0.851), *P* < 0.0001). ACT: adjuvant chemotherapy; OS: overall survival; and non-ACT: nonadjuvant chemotherapy.

**Figure 2:**
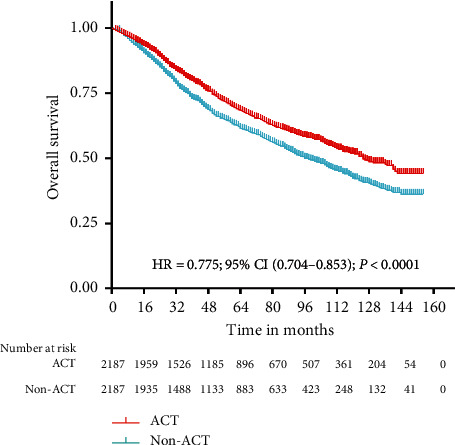
Overall survival for the matched study cohort. The Kaplan–Meier survival analysis for matched cohort showed that ACT had better OS than non-ACT (HR = 0.775, CI: (0.704–0.853), *P* < 0.0001). ACT: adjuvant chemotherapy; OS: overall survival; and non-ACT: nonadjuvant chemotherapy.

The result of Kaplan–Meier survival analysis revealed that ACT achieved better OS than non-ACT in the entire study cohort (HR = 0.800, CI: (0.751–0.851), *P* < 0.0001) (Figure 1). After propensity-score matching, there were 2,187 patients in each group. All baseline characteristics were well matched, with standardized differences for all variables of 3% or less (Table 2). In the matched analyses, ACT also showed better OS than non-ACT (HR = 0.775, CI: (0.704–0.853), *P* < 0.0001) (Figure 2).

### 3.3. Independent Prognostic Factors in Non-ACT Group

Univariate and multivariate Cox regression analysis presented that eight prognostic factors, including gender, age, grade, pathological subtype, VPI, tumor size, surgical procedure, and the number of removed lymph nodes, were significantly correlated with OS in the non-ACT group (Table 3).

### 3.4. Prognostic Nomogram Establishment for Overall Survival and Predictive Performance

**Figure 3:**
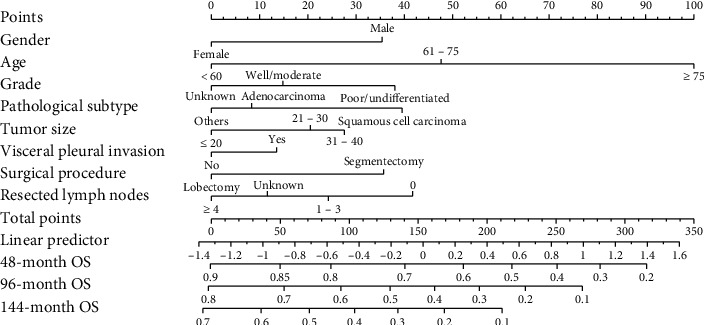
The established nomogram model for predicting 48-, 96-, and 144-month OS in NSCLC stage IB patients after surgical resection. OS: overall survival and NSCLC: non-small-cell lung cancer.

**Figure 4:**
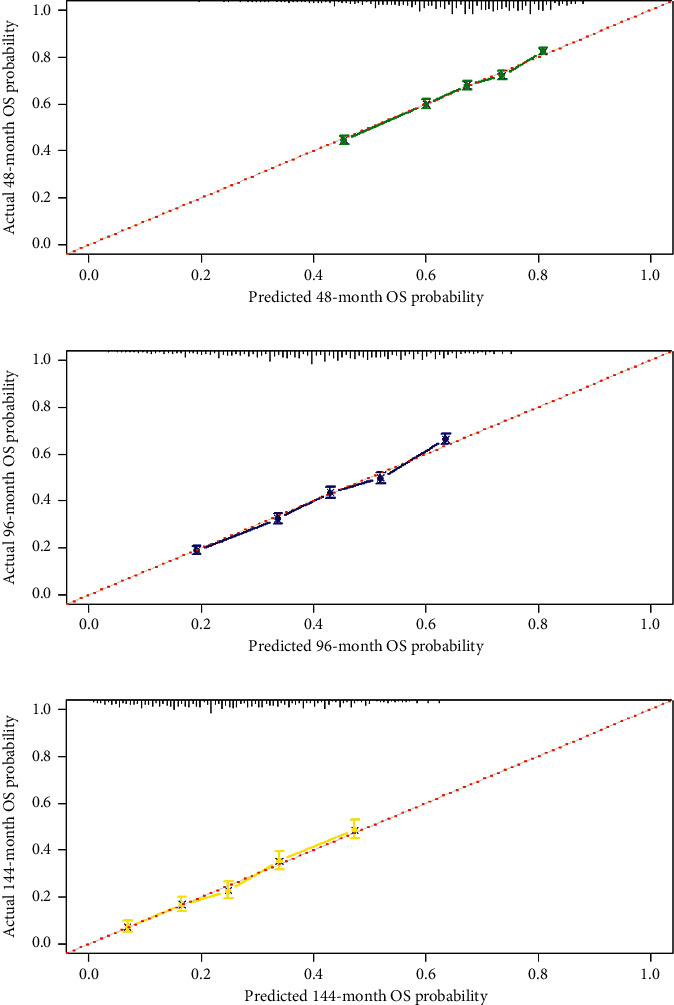
Calibration curves for predicting patient survival at each time. (a) 48-month OS, (b) 96-month OS, and (c) 144-month OS. The results showed good agreement between prediction and observation in the probability of 48-, 96-, and 144-month OS. OS: overall survival.

Based on the results of multivariate Cox regression analysis, the nomogram for OS prediction was formulated (Figure 3). In nomogram, eight prognostic factors (gender, age, grade, pathological subtype, VPI, tumor size, surgical procedure, and resected lymph nodes) were assigned a score. The 48-, 96-, and 144-month probability of OS was calculated for specific patient by accumulating the score. The C-index for prediction of OS was 0.639 (95% CI: 0.632–0.646). The Hosmer–Lemeshow test showed that there was no significant deviation between the observed and predicted OS (*P*=0.49). The calibration curve also showed good congruence between the predicted probability of OS and actually observed probability of OS (Figure 4).

### 3.5. Subgroup Analysis to Select Adjuvant Chemotherapy Candidates

To further evaluate the influence of tumor size on the selection of potential ACT candidates for patients with stage IB NSCLC, subgroup analyses were executed. Results from multivariate Cox regression analysis of tumor size revealed that ACT shared similar OS to non-ACT in NSCLC patients with tumor size ≤20 mm (HR = 1.068, 95% CI (0.919–1.241), *P*=0.394) (Table 4). However, for NSCLC patients with 20 mm < size ≤30 mm (HR = 0.845, 95% CI (0.724–0.986), *P*=0.032) and 30 mm < size ≤ 40 mm (HR = 0.912, 95% CI (0.833–1.000), *P*=0.049), ACT is related to better OS (Table 4).

## 4. Discussion

Tumor relapse and distant metastasis postoperation are a leading cause to reduce OS for patients with IB stage NSCLC. According to 7th edition TNM staging system, stage IB consists of tumors >4 cm and ACT has showed promise to reduce the incidence of recurrent or metastatic tumors for those patients [23, 24]. However, 8th edition TNM staging system puts tumors >4 cm into stage IIA [6]. So, theoretically, most patients with stage IB NSCLC might not benefit from ACT now. However, Wang et al. compared survival outcomes between the 8th and 7th editions TNM staging system for patients with stage IB NSCLC, and they found stage IB patients with good performance status based on 8th editions of the AJCC TNM staging system also can benefit from ACT [25]. A meta-analysis also showed that ACT after surgery was beneficial to OS and progression-free survival in stage IB NSCLC patients [26]. Other studies found different results that ACT is not correlated with improved survival [27, 28]. Thus, there is no consensus on the ACT of current stage IB NSCLC. In this study, we analyzed the efficacy of ACT in stage IB NSCLC patients based on 8th edition TNM staging system classification. Our study population was from SEER database, and numerous high-quality studies have used data from SEER for survival analyses [29, 30]. We found that ACT achieved better OS than non-ACT.

Some studies proposed that stage IB NSCLC patients with high-risk factors should receive ACT [31, 32]. According to the current NCCN guidelines, poorly differentiated tumors, vascular invasion, VPI, unknown lymph node status, and wedge resection have been listed as high-risk factors in patients with stage IB NSCLC. However, the impact of the existence of a single or several high-risk factors on the choice of ACT remains unclear. Many studies have tried to explore the association between risk factors and outcome. Mohit et al. found that age, gender, tumor size, and surgical procedure are important prognostic factors for survival in patients with early stage (I and II) NSCLC after surgical resection [33]. Liu et al. reported that poor differentiation is related to prognosis in pathological surgically treated stage I NSCLC and can be an independent prognostic factor [34]. A previous study confirmed the number of LNs evaluated during surgery is associated with NSCLC patient's survival after surgical resection [35]. Although these studies analyzed the impact of some risk factors in prognosis, their results were not applicable to clinical practice. In this study, we identified gender, age, grade, pathological subtype, VPI, tumor size, surgical procedure, and the number of removed lymph nodes as independent prognostic factors for OS. Compared with their results and several previous studies, our results were more comprehensive [36, 37]. Moreover, in order to solute the problem of clinical applicability, we built prognostic model for clinician. In this study, we incorporated most recognized risk factors (gender, age, grade, pathological subtype, tumor size, VPI, surgical procedure, and the number of removed lymph nodes) to formulate a risk prediction nomogram for OS prediction by using a large multicenter population. The C-index of the conventional TNM staging system was 0.596 (95% CI: 0.551–0.641) [38]. For our nomogram, the C-index was 0.639 (95% CI: 0.632–0.646). So, our nomogram presented better predictive accuracy of overall survival than the conventional TNM staging system. In the internal cohort, the Hosmer–Lemeshow test and calibration showed good congruence between the predicted probability of OS and actually observed probability of OS. Taken together, our nomogram has sufficient credibility to predict OS in postoperative IB stage patients. What is more, because all factors in our nomogram were the existing clinical data, clinician can easily conduct an individualized survival prediction for postoperative stage IB NSCLC patients in clinical practice. So, this model would facilitate clinician's better decisions on the application of adjuvant chemotherapy.

In subgroups, the results from multivariate analyses of tumor size revealed that ACT shared similar OS to non-ACT in NSCLC patients with tumor size ≤20 mm. However, for NSCLC patients with 20 mm < size ≤30 mm and 30 mm < size ≤40 mm, ACT is associated with better OS. In postoperative lung cancer patients with no nodal spread, the 5-year survival rate declines with increasing tumor size: 3 to 4 cm, 74%; 4 to 5 cm, 65%; and 5 to 7 cm, 57% [39]. We acknowledged that ACT was likely to be a choice for younger patients who suffered a larger tumor size. American Society of Clinical Oncology/Cancer Care Ontario Clinical Practice Guideline also proposed that benefits of ACT outweigh harms in NSCLC stage IB patients with larger tumors [11]. Above all, for those stage IB NSCLC patients with tumor size >20 mm and ≤40 mm, ACT may be a good choice, whereas multicenter randomized controlled trials (RCTs) need to validate our conclusion.

Our study still has some deficiencies. First, there are numerous types of immunotherapies, and targeted therapies for adjuvant therapy in NSCLC stage IB have increasingly been explored in clinical trials. The results from these studies may be greatly different with ACT, whereas impacts of adjuvant therapy used various immunotherapies and targeted therapies on OS in patients which could not be published now. Furthermore, the indication of targeted therapy and immunotherapy is rigorous, and the cost is so expensive that many patients cannot afford. By all accounts, their results may not substantially affect our conclusion. Second, nomogram should be clarified through external validation. So, many well-designed study cohorts in the future are needed to further validate our conclusions. Third, due to the lack of information about ACT regimen and LVI, we were unable to evaluate the influence of ACT regimen and LVI on OS. Fourth, similar to other SEER-based studies, the data were obtained from public databases and thus some information, such as chemotherapy data, may not be accurate. What is more, the SEER database lacks some information, such as surgical quality and R0 margin. These treatments can significantly improve patient outcomes and may skew the conclusions. Finally, some bias was inevitable due to the nature of a retrospective study.

Despite these limitations, the results presented in this study provided a predictive nomogram to predict OS for patients with 8th edition stage IB NSCLC and firstly proposed ACT candidates for stage IB patients based on tumor size. They might also serve as stratification tools for clinical studies and provide evidence for the development of interventions aimed at improving overall survival.

## 5. Conclusion

We observed that ACT achieved better OS than non-ACT and found that eight prognostic factors, including gender, age, grade, pathological subtype, VPI, tumor size, surgical procedure, and the number of removed lymph nodes, were significantly correlated with OS in postoperative NSCLC stage IB patients. We further formulated a nomogram to support individualized assessment of OS in NSCLC stage IB patients after surgical resection. The subsequent subgroup analysis confirmed that patients with tumor size >20 mm and ≤40 mm may be potential candidates for ACT.

## Figures and Tables

**Table 1:** Baseline characteristics of the study population.

	Nonadjuvant chemotherapy (*n* = 14578)	Adjuvant chemotherapy (*n* = 2187)		*P* value
Gender				0.099
Male	6961 (47.8%)	1003 (45.9%)		
Female	7617 (52.2%)	1184 (54.1%)		

Age				<0.001
<60	2090 (14.3%)	564 (25.8%)		
61–75	7489 (51.4%)	1322 (60.4%)		
≥75	4999 (34.3%)	301 (13.8%)		

Race				0.533
White	12372 (84.9%)	1836 (84.0%)		
Black	1184 (8.1%)	187 (8.6%)		
Others	1022 (7.0%)	164 (7.4%)		

Marital status				<0.001
Divorced	1649 (11.3%)	266 (12.2%)		
Married	8193 (56.2%)	1328 (60.7%)		
Others	4736 (32.5%)	593 (27.1%)		

Laterality				0.005
Left	6059 (41.6%)	840 (38.4%)		
Right	8519 (58.4%)	1347 (61.6%)		

Grade				<0.001
Well/moderate	8729 (59.9%)	1132 (51.8%)		
Poor/undifferentiated	4919 (33.7%)	935 (42.7%)		
Unknown	930 (6.4%)	120 (5.5%)		

Pathological subtype				<0.001
Adenocarcinoma	6103 (41.9%)	1027 (47.0%)		
Squamous cell carcinoma	3581 (24.5%)	411 (18.8%)		
Others	4894 (33.6%)	749 (34.2%)		

Visceral pleural invasion				0.452
No	5869 (40.3%)	862 (39.4%)		
Yes	8709 (59.7%)	1325 (60.6%)		

Tumor size (mm)				0.002
≤20	3494 (24.0%)	470 (21.5%)		
21–30	3355 (23.0%)	472 (21.6%)		
31–40	7729 (53.0%)	1245 (56.9%)		

Surgical procedure				0.005
Lobectomy	11723 (80.4%)	1814 (82.9%)		
Segmentectomy	2855 (19.6%)	373 (17.1%)		

No. of resected lymph nodes				0.938
0	1442 (9.9%)	214 (9.8%)		
1–3	2541 (17.4%)	370 (16.9%)		
≥4	9877 (67.7%)	1495 (68.4%)		
Unknown	718 (4.9%)	108 (4.9%)		

**Table 2:** Baseline characteristics of the propensity-score matched population.

	Nonadjuvant chemotherapy (*n* = 2187)	Adjuvant chemotherapy (*n* = 2187)	*P* value	Standardized difference
Gender			0.952	0
Male	1005 (46.0%)	1003 (45.9%)		
Female	1182 (54.0%)	1184 (54.1%)		

Age			0.887	<0.001
<60	576 (26.3%)	564 (25.8%)		
61–75	1308 (59.8%)	1322 (60.4%)		
≥75	303 (13.9%)	301 (13.8%)		

Race			0.678	0.030
White	1855 (84.8%)	1836 (84.0%)		
Black	181 (8.3%)	187 (8.6%)		
Others	151 (6.9%)	164 (7.4%)		

Marital status			0.682	−0.022
Divorced	249 (11.4%)	266 (12.2%)		
Married	1333 (59.9%)	1328 (60.7%)		
Others	605 (27.7%)	593 (27.1%)		

Laterality			0.950	0.003
Left	842 (38.5%)	840 (38.4%)		
Right	1345 (61.5%)	1347 (61.6%)		

Grade			0.598	0.023
Well/moderate	1163 (53.2%)	1132 (51.8%)		
Poor/undifferentiated	903 (41.3%)	935 (42.7%)		
Unknown	121 (5.5%)	120 (5.5%)		

Pathological subtype			0.845	0.017
Adenocarcinoma	1045 (47.8%)	1027 (47.0%)		
Squamous cell carcinoma	407 (18.6%)	411 (18.8%)		
Others	735 (33.6%)	749 (34.2%)		

Visceral pleural invasion			0.829	<0.001
No	869 (39.7%)	862 (39.4%)		
Yes	1318 (60.3%)	1325 (60.6%)		

Tumor size (mm)			0.994	<0.001
≤20	469 (21.4%)	470 (21.5%)		
21–30	475 (21.7%)	472 (21.6%)		
31–40	1243 (56.9%)	1245 (56.9%)		

Surgical procedure			0.686	0.013
Lobectomy	1823 (83.3%)	1814 (82.9%)		
Segmentectomy	364 (16.7%)	373 (17.1%)		

No. of resected lymph nodes			0.288	−0.023
0	182 (8.3%)	214 (9.8%)		
1–3	388 (17.7%)	370 (16.9%)		
≥4	1518 (69.4%)	1495 (68.4%)		
Unknown	99 (4.6%)	108 (4.9%)		

**Table 3:** Univariable and multivariable analysis for OS using the Cox proportional hazard model in the non-ACT group.

	Univariate analysis			Multivariate analysis		
	HR	95%C	*P*	HR	95%C	*P*
Gender						
Male	1			1		
Female	0.699	0.667–0.734	<0.001^*∗*^	0.701	0.666–0.737	<0.001^*∗*^

Age						
<60	1			1		
61–75	1.634	1.499–1.781	<0.001^*∗*^	1.504	1.379–1.641	<0.001^*∗*^
≥75	2.659	2.438–2.899	<0.001^*∗*^	2.329	2.132–2.544	<0.001^*∗*^

Race						
White	1			1		
Black	0.861	0.785–0.945	0.002^*∗*^	0.931	0.848–1.022	0.134
Others	0.765	0.690–0.848	<0.001^*∗*^	0.853	0.768–0.946	0.003^*∗*^

Marital status						
Divorced	1			1		
Married	0.922	0.853–0.997	0.042^*∗*^	0.822	0.759–0.890	<0.001^*∗*^
Others	1.071	0.987–1.163	0.100	0.990	0.911–1.076	0.816

Laterality						
Left	1			—		
Right	0.966	0.920–1.014	0.162	1.002	0.955–1.052	0.933

Grade						
Well/moderate	1			1		
Poor/undifferentiated	1.313	1.249–1.380	<0.001^*∗*^	1.216	1.155–1.279	<0.001^*∗*^
Unknown	0.784	0.703–0.874	<0.001^*∗*^	0.886	0.792–0.990	0.032^*∗*^

Pathological subtype						
Adenocarcinoma	1			1		
Squamous cell carcinoma	1.480	1.397–1.567	<0.001^*∗*^	1.285	1.210–1.364	<0.001^*∗*^
Others	0.903	0.853–0.957	0.001^*∗*^	0.929	0.875–0.985	0.014^*∗*^

Visceral pleural invasion						
No	1			1		
Yes	1.041	0.992–1.093	0.103	1.127	1.045–1.214	0.002^*∗*^

Tumor size (mm)						
≤20	1			1		
21–30	1.171	1.092–1.257	<0.001^*∗*^	1.195	1.112–1.284	<0.001^*∗*^
31–40	1.118	1.053–1.118	<0.001^*∗*^	1.269	1.167–1.380	<0.001^*∗*^

Surgical procedure						
Lobectomy	1			1		
Segmentectomy	1.653	1.563–1.749	<0.001^*∗*^	1.350	1.258–1.449	<0.001^*∗*^

No. of resected lymph nodes						
0	1			1		
1–3	0.699	0.643–0.760	<0.001^*∗*^	0.858	0.783–0.941	0.001^*∗*^
≥4	0.539	0.502–0.579	<0.001^*∗*^	0.700	0.641–0.764	<0.001^*∗*^
Unknown	0.621	0.549–0.702	<0.001^*∗*^	0.771	0.678–0.878	<0.001^*∗*^

^*∗*^The difference was statistically significant. OS: overall survival; ACT: adjuvant chemotherapy; HR: hazard ratio; and CI: confidence interval.

**Table 4:** Subgroup analyses stratified by tumor size for overall survival.

	≤20 mm			21 mm–30 mm			31 mm–40 mm		
	HR	95%CI	*P*	HR	95%CI	*P*	HR	95%CI	*P*
Age									
<60	1			1			1		
61–75	1.343	1.153–1.563	<0.001	1.675	1.407–1.993	<0.001	1.479	1.333–1.640	<0.001
≥75	1.927	1.641–2.264	<0.001	2.525	2.112–3.018	<0.001	2.335	2.097–2.600	<0.001

Gender									
Male	1			1			1		
Female	0.669	0.606–0.739	<0.001	0.725	0.657–0.801	<0.001	0.722	0.678–0.770	<0.001

History of chemotherapy									
Nonadjuvant chemotherapy	1			1			1		
Adjuvant chemotherapy	1.068	0.919–1.241	0.394	0.845	0.724–0.986	0.032	0.912	0.833–1.000	0.049

## Data Availability

All data and materials analyzed during this study are available from the SEER database.

## Abbreviations

ACT: Adjuvant chemotherapy
NSCLC: Non-small-cell lung cancer
SEER: Surveillance, Epidemiology, and End Results
NCCN: National Comprehensive Cancer Network
OS: Overall survival
C-index: Concordance index
VPI: Visceral pleural invasion
HR: Hazard ratio
CI: Confidence interval
LNs: Lymph nodes.
